# Patients with anti-PM/Scl-positive and idiopathic inflammatory myopathy resemble anti-synthetase syndrome

**DOI:** 10.1186/s42358-025-00441-y

**Published:** 2025-02-14

**Authors:** Rafaella do Amaral Barbosa, Samuel Katsuyuki Shinjo

**Affiliations:** 1 Division of Rheumatology, Faculdade de Medicina FMUSP, Universidade de Sao Paulo, Sao Paulo, SP Brazil; 2 Division of Rheumatology, Faculdade de Medicina, Universidade de Sao Paulo, Av. Dr. Arnaldo, 455, 3º andar, sala 3184 - Cerqueira César, Sao Paulo, SP CEP 01246-903 Brazil

**Keywords:** Anti-PM/Scl, Autoantibodies, Phenotypic, Systemic autoimmune myopathies, Inflammatory myopathies, Myositis

## Abstract

**Background:**

Anti-PM/Scl autoantibody has been associated with an overlap between polymyositis (PM) and systemic sclerosis (SSc). However, due to limited studies, the relevance of this autoantibody in patients with idiopathic inflammatory myopathies (IIMs) without SSc was analyzed.

**Methods:**

This single-center retrospective cohort study was conducted between 2004 and 2024. A total of 93 adult patients with IIMs (66 with dermatomyositis and 27 with PM - EULAR/ACR 2017) without SSc were included: 16 anti-PM/Scl(+) and 77 anti-PM/Scl(-). Patients with other types of IIMs, cancer-associated myositis, or overlap myositis, including SSc, as well as those with other myositis-specific and/or myositis-associated autoantibodies were excluded.

**Results:**

The median age, sex distribution, and median follow-up duration were comparable between the anti-PM/Scl(+) and anti-PM/Scl(-) groups. There were no differences in clinical and laboratory characteristics, except for a higher frequency of lung involvement, joint involvement, “mechanics’ hand,” “hiker’s feet,” and Raynaud’s phenomenon, in contrast to a lower frequency of facial rash and “V”-neck sign in patients with anti-PM/Scl(+) than in those with anti-PM/Scl(-) (all *P* < 0.05). Furthermore, patients with anti-PM/Scl(+) exhibited a higher frequency of disease relapse (68.8% vs. 33.8%), disease activity (50.0% vs. 24.7%), and immunosuppressant use (methotrexate or azathioprine) at the last medical evaluation (all *P* < 0.05). Severe infection and death rates were comparable between the groups.

**Conclusions:**

Anti-PM/Scl positivity was observed in 17.2% of the sample analyzed in the present study. Patients with this autoantibody present clinical manifestations resembling anti-synthetase syndrome, with increased disease relapse and activity rates.

## Introduction

Idiopathic inflammatory myopathies (IIMs) or systemic autoimmune myopathies constitute a group of rare rheumatic diseases characterized by inflammatory features that predominantly affect skeletal musculature [1, 2]. Based on demographics, evolutionary and histological characteristics, including systemic manifestations, IIMs can be classified as dermatomyositis (DM), polymyositis (PM), clinically amyopathic dermatomyositis (CADM), immune-mediated necrotizing myopathy (IMNM), anti-synthetase syndrome (ASyS), inclusion body myositis (IBM), among others [1–3].

Various autoantibodies are associated with IIMs. These can be classified as myositis-specific or myositis-associated autoantibodies [1]. Among the myositis-associated autoantibodies, anti-PM/Scl has been observed in patients with an overlap between autoimmune myopathy (for example, PM) and systemic sclerosis (SSc) [4–11].

However, limited are available in the literature, primarily case reports and series, including patients affected by IIMs with anti-PM/Scl autoantibodies without systemic sclerosis. These patients reportedly exhibit joint and/or pulmonary involvement [4, 12–24], “hiker’s feet” [20, 24–26], esophageal involvement [15, 18, 23, 27] and/or Raynaud’s phenomenon [15, 18, 24]. Furthermore, as limitations, these studies defined patients with IIMs according to the Bohan and Peter criteria [13, 15, 17, 21, 23, 28, 29], did not simultaneously analyze the presence of other myositis-specific or myositis-associated autoantibodies [13, 15–18, 21] and/or did not exclude patients with others systemic autoimmune diseases, such as Sjögren’s syndrome [20, 23, 28] and systemic sclerosis [14, 16, 20, 23, 28, 29].

Therefore, the present study aimed to evaluate the isolated significance of anti-PM/Scl autoantibodies in a representative sample of patients with IIMs (DM or PM) without systemic sclerosis, other systemic autoimmune diseases or other myositis-specific and myositis-associated autoantibodies. In addition, patients were assessed for demographic, clinical, laboratory, therapeutic, evolutionary, and prognostic parameters.

## Patients and methods

This study was a retrospective cohort single-center investigation conducted from 2004 to 2024, encompassing adult patients with IIMs (DM or PM) from the inflammatory myopathy outpatient clinic of a tertiary center.

Inclusion criteria. Adult patients with IIMs according to the European League Against Rheumatism/American College of Rheumatology (EULAR/ACR) 2017 classification criteria [30]; availability of blood samples for myositis-specific and myositis-associated autoantibody tests, including anti-PM/Scl.

Exclusion criteria. Patients with IIMs (DM or PM) associated with other systemic autoimmune diseases (rheumatoid arthritis, systemic sclerosis, systemic lupus erythematosus, or Sjögren’s disease); patients with IMNM, CADM, IBM, and myositis associated with neoplasms; patients with any positive myositis-specific and myositis-associated autoantibodies positivity, except those with anti-PM/Scl(+).

Control group. Adult patients with IIMs according to the European League Against Rheumatism/American College of Rheumatology (EULAR/ACR) 2017 classification criteria [30] and negative for myositis-specific and myositis-associated autoantibodies, including anti-PM/Scl.

The following data from eligible patients (anti-PM/Scl cases and control group) were collected from electronic medical records with pre-standardized and parameterized information:

1. Demographics: Current age, age at disease diagnosis, sex, and ethnicity;
2. Clinical manifestations: Symptoms onset, follow-up time, cutaneous involvement (heliotrope, facial *rash*, Gottron’s sign/papules, “V-neck” sign, “shawl” sign, “mechanic’s hand,” “hiker’s feet,” periungual hyperemia, vasculopathy, ulcers and calcinosis), Raynaud’s phenomenon, systemic manifestations (dysphagia, arthritis, dyspnea, weight loss and fever), muscle weakness of upper and lower limbs graded according the Medical Research Council (MRC) [31]; medications prescribed at the first and last appointments and their respective doses;
3. Laboratory: Serum levels of muscle enzymes [creatine phosphokinase (CPK), aspartate aminotransferase (AST), alanine aminotransferase (ALT), and lactic dehydrogenase (LDH)], and antinuclear antibodies (ANA) in blood samples;
4. Disease status. Disease activity status (based on the last medical appointment): clinical remission (no evidence of disease activity for at least six months without DM treatment), complete clinical response (no evidence of disease activity for at least six months, whereas still receiving myositis therapy), and disease relapse: recurrence of clinical (muscle or skin manifestations) and/or laboratory findings (elevated creatine phosphokinase) with no explanation other than disease activity;
5. Complementary examinations: Changes in high-resolution computer tomography images of the lung, indicating interstitial lung disease (ILD). The ILD was defined as the presence of ground-glass opacities, reticular pattern, honeycombing, traction bronchiectasis, or fibrosis;
6. An analysis of myositis-specific and myositis-associated autoantibodies was performed in patients’ serum samples stored at − 20ºC. These biological samples were collected at the time of the initial patient follow-up and after obtaining informed and voluntary consent from the patients.

Anti-PM/Scl, -OJ, -EJ, -PL-7, -PL-12, -SRP, -Jo-1, -SAE-1, -NXP-2, -MDA-5, -TIF-1γ, -Mi-2, -Ro-52, and -Ku autoantibodies were tested using a commercial kit (Euroimmun, Lübeck, Germany), according to the manufacturer’s protocol. The evaluation of results was based on method established in a previous study [32].

### Statistical analysis

The Kolmogorov-Smirnov test was used to evaluate the distribution of continuous variables. Results are presented as median (interquartile range: 75th -25th ) or frequency (%). Comparisons between the anti-PM/Scl-positive and anti-PM/Scl-negative groups were conducted using the Student’s t-test or Mann-Whitney U test for continuous variables and the chi-square test or Fisher’s exact test for categorical variables. Statistical significance was set at *P* < 0.05. All analyses were performed using SPSS Statistics, version 15.0 (Chicago, IL, USA).

## Results

**Fig. 1:**
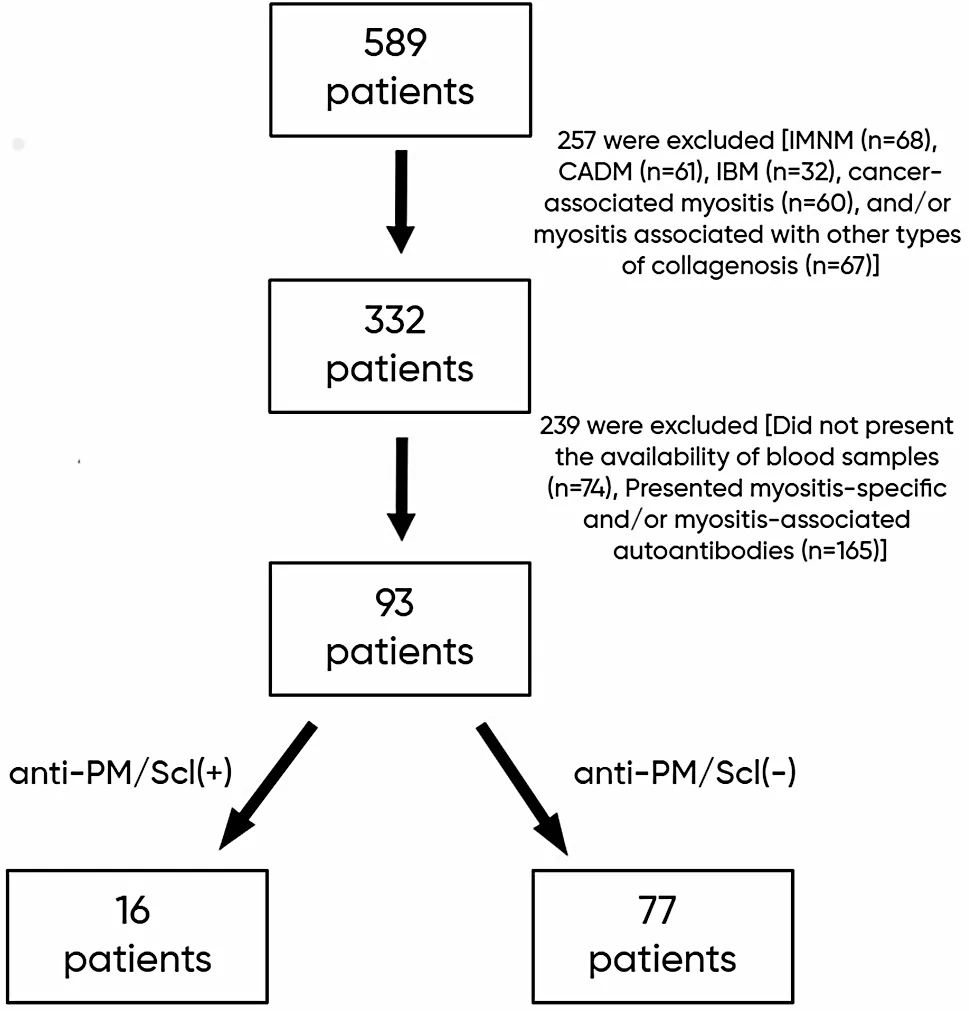
Selection flowchart

Of the 589 adult patients with IIMs (DM or PM), 257 were excluded due to presenting IMNM (*n* = 68), CADM (*n* = 61), IBM (*n* = 32), cancer-associated myositis (*n* = 60), and/or myositis associated with other types of rheumatic diseases (*n* = 67). Some patients exhibited overlap of more than one type of rheumatic diseases and/or neoplasm. Furthermore, 74 cases were excluded due to the unavailability of blood samples for autoantibodies analysis and 165 cases were excluded because they presented myositis-specific (44 with anti-Jo-1, 32 with anti-MDA-5, 18 with anti-Mi-2, 14 with anti-PL-7, 12 with anti-PL-12, 10 with anti-TIF-1γ, seven with anti-NXP-2, seven with anti-SAE, six with anti-EJ, and/or four with anti-SRP) and/or myositis-associated autoantibodies (74 with anti-Ro-52 or 12 with anti-Ku). Some patients tested positive for more than one autoantibody (Fig. 1).

Of the remaining 93 patients, 16 (17.2%) presented with only anti-PM/Scl(+) and 77 (82.8%) with anti-PM/Scl(-) (control group), of which 66 and 27 patients were initially diagnosed with DM and PM, respectively.

Age, sex, and ethnicity distributions were comparable between the anti-PM/Scl(+) and anti-PM/Scl(-) groups (Table 1).

**Table 1:** General characteristics of adult patients with idiopathic inflammatory myopathies, according to presence or absence of anti-PM/Scl autoantibodies

Characteristics	Anti-PM/Scl(+) (*n* = 16)	Anti-PM/Scl(-) (*n* = 77)	*p* value
Age at disease diagnosis (years)	42 (29–59)	41 (31–53)	0.607
Female sex	13 (81.3)	60 (77.9)	> 0.999
White ethnicity	14 (87.5)	57 (74.0)	0.342
Initial disease diagnosis			
Dermatomyositis	11 (68.8)	55 (71.4)	> 0.999
Polymyositis	5 (31.2)	22 (28.6)	> 0.999
Fever	3 (18.8)	17 (22.1)	> 0.999
Weight loss	9 (56.2)	33 (42.9)	0.411
Gottron’s sign / papules	10 (62.4)	48 (62.3)	> 0.999
Heliotrope rash	7 (43.8)	46 (59.7)	0.276
Facial rash	2 (12.5)	33 (42.9)	0.025
“V”-neck sign	1 (6.3)	33 (42.9)	0.005
“Shawl” sign	0	21 (27.3)	-
Periungual hyperemia	8 (50.0)	30 (39.0)	0.420
Raynaud’s phenomenon	11 (68.8)	29 (37.7)	0.028
Vasculopathy	0	14 (18.2)	-
“Mechanics’ hands”	11 (68.8)	17 (22.1)	0.001
“Hiker’s feet”	8 (50.0)	5 (6.5)	< 0.001
Skin ulcers	2 (12.5)	9 (11.7)	> 0.999
Calcinosis	0	4 (5.2)	-
Muscle strength (MRC)			
Upper limbs			
V degree	3 (18.8)	16 (20.8)	> 0.999
IV degree	10 (62.4)	44 (57.1)	
III degree	3 (18.8)	14 (18.2)	
II degree	0	3 (3.9)	
Lower limbs			
V degree	0	16 (20.8)	> 0.999
IV degree	14 (87.5)	42 (54.5)	
III degree	2 (12.5)	18 (23.4)	
II degree	0	1 (1.3)	
Dysphagia	7 (43.8)	30 (39.0)	0.784
Arthritis	10 (62.4)	24 (31.2)	0.024
Lung involvement			
Dyspnea	10 (62.4)	27 (35.1)	0.005
Interstitial pneumopathy	14 (87.5)	25 (32.5)	< 0.001
Ground glass opacities	11 (68.8)	21 (27.3)	0.003
Fibrosis	8 (50.0)	11 (14.3)	0.003
Maximum levels of muscle enzymes			
Creatine phosphokinase (U/L)	544 (202–3819)	277 (67-1748)	0.228
Aspartate aminotransferase (U/L)	45 (28–155)	47 (27–131)	0.891
Alanine aminotransferase (U/L)	40 (28–121)	40 (24–133)	0.935
Lactic dehydrogenase (U/L)	545 (350–867)	592 (372–833)	0.695
Antinuclear antibodies	16 (100)	55 (71.4)	0.034

Data are expressed as median (IQR 25th − 75th ) or percentage (%)

MRC: Medical Research Council

Clinical manifestations were also similar between the groups, except for a higher frequency of lung and joint involvements, “mechanics’ hand,” “hiker’s feet,” and Raynaud’s phenomenon; and a lower frequency of facial rash and “V”-neck sign in patients with anti-PM/Scl(+) compared to anti-PM/Scl(-) patients.

Serum levels of muscle enzymes were comparable between the groups; however, a higher frequency of antinuclear antibodies was observed in patients with anti-PM/Scl(+) than in those with anti-PM/Scl(-).

The follow-up durations were similar between the groups (Table 2). During follow-up, there was no significant difference in the infection and mortality rates between the groups, except for disease relapse, number of individuals with active disease, and disease remission rates (at the last medical evaluation) among the patients with anti-PM/Scl(+).

**Table 2:** Treatment characteristics and follow-up of 93 patients with idiopathic inflammatory myopathies

Characteristics	Anti-PM/Scl(+) (*n* = 16)	Anti-PM/Scl(-) (*n* = 77)	*p* value
Current follow-up			
Duration of follow-up (months)	94 (20–140)	64 (65–93)	0.244
Infections	2 (12.5)	21 (27.3)	0.341
Relapses	11 (68.8)	26 (33.8)	0.023
Death	1 (6.3)	1 (1.3)	0.402
Disease status at the last medical evaluation			
Active disease (clinical relapse)	8 (50.0)	19 (24.7)	0.025
Complete clinical response	6 (37.5)	21 (27.3)	
Disease remission	2 (12.5)	37(48.0)	
Treatment			
Previous IVMP pulse therapy	5 (31.2)	12 (15.6)	0.235
Previous IVIg	0	5 (6.5)	-
Previous IVIg + IVMP pulse therapy	3 (18.8)	21 (27.3)	0.478
No IVMP or IVIg	8 (50.0)	39 (50.6)	0.962
Current treatment			
Glucocorticoid			
Current use	7 (43.8)	21 (27.3)	0.134
Current dose (mg/day)	10 (5–20)	10 (5–20)	0.534
Methotrexate	6 (37.5)	10 (13.0)	0.027
Azathioprine	5 (31.2)	8 (10.4)	0.029
Mycophenolate mofetil	4 (25.0)	13 (16.9)	0.696
Leflunomide	0	4 (5.2)	-
Cyclosporine	5 (31.2)	22 (28.6)	0.823
Cyclophosphamide	3 (18.8)	7 (9.1)	0.256
Rituximab	4 (25.0)	8 (10.4)	0.113

Data are expressed as median (IQR 25th − 75th ) or percentage (%)

IVIg: intravenous immunoglobulin; IVMP: intravenous methylprednisolone

Previous treatments were comparable between the analyzed groups, anti-PM/Scl(+) and anti-PM/Scl(-), as well as the medical treatment established at the last medical evaluation, except for a higher use of methotrexate and azathioprine in patients with anti-PM/Scl(+).

## Discussion

In the present sample, the isolated presence of anti-PM/Scl autoantibodies in patients with IIMs (DM or PM) without systemic sclerosis was 17.2%. Compared to patients who were anti-PM/Scl-negative, those with anti-PM/Scl positivity exhibited higher frequencies of lung and joint involvements, “mechanics’ hands,” “hiker’s feet” and Raynaud’s phenomenon, as well as higher rates of relapse and lower rates of remission. Furthermore, these patients presented a higher frequency of disease activity and increased use of immunosuppressants (methotrexate or azathioprine) during the last medical evaluation.

In contrast to the studies available in the literature [13–18, 20–21, 23, 28, 29], the present study evaluated the presence of anti-PM/Scl in a homogeneous and representative sample of patients, without associated systemic autoimmune diseases or the presence of any other myositis-specific and myositis-associated autoantibodies. Conversely, prior studies which analyzed the anti-PM/Scl primarily consisted in cases reports or series [13, 17, 19–20, 23–27], in addition to not having excluded the possible presence of others myositis-specific and myositis-associated autoantibodies [13, 15–18, 21, 29] or others systemic autoimmune diseases [14, 16, 23, 28, 29]. These parameters can be confounding factors to a probable joint or lung manifestations, in addition to the presence of Raynaud’s phenomenon in the patients of these studies [13, 14, 16–18, 21, 23, 28,29].

Furthermore, the studies available in the literature often relied on the Bohan and Peter classification criteria, which have lower specificity for PM diagnosis compared to the EULAR/ACR 2017 criteria used in the current study. Moreover, cases of CADM, cancer-associated myositis, and IMNM were excluded, ensuring that only patients with DM or PM were included.

When compared to our patients with anti-PM/Scl(-), those with anti-PM/Scl(+) exhibited clinical manifestations similar to ASyS, such as Raynaud’s phenomenon, “mechanics’ hands,” “hiker’s feet,” joints, and lung involvement. This is consistent with previous studies, where, more than 60% of the patients with anti-PM/Scl(+) presented Raynaud phenomenon [4, 12–13, 18, 21, 24], and over 75% of patients manifested interstitial lung disease [4, 12, 14, 16–17, 20, 24]. Other few studies [3, 20, 22] reported the presence of “Hiker’s feet” in over 80% of the analyzed cases.

Li et al. [33] evaluated 97 patients with DM and without myositis-specific autoantibodies (anti-Mi-2, -TIF-1γ, -MDA-5, -NXP-2, -SAE-1, -SRP, -Jo-1, -PL-7, -PL-12, -EJ, -OJ, and -HMGCR). Subsequently, these patients were divided into two clusters, one of which exhibited a higher frequency of lung involvement and, consequently, of “mechanic’s hands,” Gottron’s sign and papules, and arthritis; therefore, they resembled the phenotypic manifestations of ASyS. The other cluster presented with less lung involvement and a significantly higher heliotrope rash rate. However, these authors [33] did not evaluate myositis-associated antibodies, including anti-PM/Scl; thus, it is plausible that in the cluster where the patients’ manifestations are similar to those with ASyS, there is, for instance, a higher prevalence of anti-PM/Scl.

Regarding follow-up, the infection rates were similar between the anti-PM/Scl-positive and anti-PM/Scl-negative groups. Among the studies available in the literature, only two evaluated the infection rates in patients with anti-PM/Scl(+) [12, 16], mainly related to hard lung infections associated with ILD [16] and a case of meningitis that evolved to death [12]. However, in the latest study [12], there was no specification if the patient had anti-PM/Scl(+). In the present study, the prevalence of infections was 12.5%, characterized pirmarily by viral and bacterial infections; however, contrary to the literature, it did not present any association with lung involvement.

Some studies [4, 9, 12, 15–18, 20, 28] have analyzed mortality rates, which ranged between 0% and 10%. In the present study, the mortality prevalence is consistent with that presented in the literature, since its value is 6.3%, with the reported cause of death being acute myocardial infarction, that is, cardiac involvement. And, despite the variety of causes of death presented in the literature (lung, infectious, cardiac, and neoplastic involvement), the cause of the death in the present study coincides with that of 2.3% of the patients in Váncsa et al.^12^ and of 5% of those in Marie et al. [15].

In addition, in the final patient evaluation, our patients with anti-PM/Scl(+) exhibited higher rates of relapses and, consequently, active disease, with increased use of immunosuppressants (methotrexate or azathioprine). Corroborating our data, Marie et al. [15] demonstrated that 12 of 20 anti-PM/Scl(+) patients, without anti-Jo-1, -PL-7, -PL-12, -Mi-2, -Ku, and -Ro-52, experienced relapses during the follow-up. Lega et al. [17], for their part, demonstrated that 44% of the patients, with anti-PM/Scl(+) and ILD experienced disease worsening or death, in the final medical evaluation, wherein all patients were receiving corticosteroids and only 44% were undergoing drug treatment in conjunction with immunosuppressants. Espinosa-Ortega et al. [34] conducted an analysis of the damage index in patients with IIM, they included patients with and without myositis-specific and myositis-associated autoantibodies. They observed that patients with anti-PM/Scl(+) demonstrated a higher rate of increase in the Myositis Damage Index (MDI) extent score per unit of time compared to other patients, indicating greater damage accrual among these patients, showing a faster progression of lung fibrosis.

A limitation of the present study is its retrospective cohort design. However, the data were collected from electronic medical records using pre-standardized and parameterized information, thereby ensuring reliability of the collected data.

## Conclusions

Anti-PM/Scl autoantibodies were observed in 17.2% of our sample patients with DM/PM, without SSc, and without other myositis-specific and myositis-associated autoantibodies. Patients with anti-PM/Scl exhibited a phenotypic pattern similar to that of ASyS, and experienced higher rates of relapse and disease activity.

## Data Availability

No datasets were generated or analysed during the current study.

## Abbreviations

ACR: American College of Rheumatology
ALT: Alanine aminotransferase
ANA: Antinuclear antibodies
ASyS: Anti-synthetase syndrome
AST: Aspartate aminotransferase
CADM: Clinically amyopathic dermatomyositis
CPK: Creatine phosphokinase
DM: Dermatomyositis
EULAR: European League Against Rheumatism
IBM: Inclusion body myositis
IIMs: Idiopathic inflammatory myopathies
ILD: Interstitial lung disease
IMNM: Immune-mediated necrotizing myopathy
IVIg: Intravenous immunoglobulin
IVMP: Intravenous methylprednisolone
LDH: Lactic dehydrogenase
MDI: Myositis Damage Index
MRC: Medical Research Council
PM: Polymyositis
SSc: Systemic sclerosis
